# Quadriceps tendon vs. patellar tendon autograft for ACL reconstruction using a hardware-free press-fit fixation technique: comparable stability, function and return-to-sport level but less donor site morbidity in athletes after 10 years

**DOI:** 10.1007/s00402-020-03508-1

**Published:** 2020-06-05

**Authors:** Alexander Barié, Thomas Sprinckstub, Jürgen Huber, Ayham Jaber

**Affiliations:** 1 Center for Orthopedics, Trauma Surgery and Spinal Cord Injury, Clinic for Orthopedics and Trauma Surgery, Heidelberg University Hospital, Schlierbacher Landstrasse 200a, 69118 Heidelberg, Germany; 2 Center for Surgery B. Nimis and Dr. T. Sprinckstub, Zur Helde 4, 69168 Wiesloch, Germany; 3 Center for Orthopedics of the Knee, Hopfenstraße 4, 69469 Weinheim, Germany

**Keywords:** Press-fit fixation, ACL reconstruction, Patellar tendon, Quadriceps autograft, Athletes

## Abstract

**Introduction:**

The use of quadriceps tendon–patellar bone (QTB) autograft for anterior cruciate ligament (ACL) reconstruction is gaining momentum. Yet, long-term results that compare this procedure with established methods are lacking. The aim of this study was to report and compare long-term results of ACL reconstruction using QTB autografts versus bone–patellar tendon–bone (BPTB) autografts, both anchored using a hardware-free press-fit fixation technique.

**Materials and methods:**

60 athletes (Tegner score ≥6) with primary ACL rupture were prospectively randomized into two groups. 56 patients were evaluated after a mean duration of 12.2 ± 1.9 months (range 10–14) and 43 patients after 10.3 ± 0.2 years (range 10–11).

**Results:**

On final follow-up, 90% of patients scored very good and good results in the functional Lysholm score (mean 99 ± 7.1, range 74–100 points). Normal or almost normal IKDC score was reported by 84% of the patients (mean 97 ± 9.5, range 60–100 points). The activity level decreased in the Tegner score from median of 7 before injury to 6 after 10 years. The KT-1000 arthrometer showed a difference in the anterior translation of less than 3 mm (mean 1.0 ± 1.2, range − 1 to 5 mm) in 91% of the patients. Significant degeneration was radiologically detected in one patient per group. No tunnel widening was seen in any patient. Up to 97% of all patients were satisfied with the operative procedure. No significant differences were found in the mentioned parameters between the two groups and also in comparison with the 1-year results. The only significant difference was in the donor site morbidity. Significantly more patients in the BPTB group had complaints during kneeling both at 1 (*p* < 0.001) and 10 years (*p* = 0.019). Squatting was also subjectively more problematic in the BPTB group than in the QTB group both after 1 (*p* = 0.003) and 10 years (*p* = 0.046).

**Conclusions:**

This study shows equally good functional, clinical and radiological long-term results for both hardware-free methods of ACL reconstruction. These results clinically confirm the safety of press-fit anchoring after 10 years. The failure rate in this study was very low, with only one re-rupture in 10 years. The increased donor site morbidity when using the BPTB autograft compared to the QTB autograft supports already reported data. It was also seen in this study for the implant-free press-fit techniques.

**Study design:**

Prospective and randomized, level of evidence 2.

## Introduction

Anterior cruciate ligament (ACL) reconstruction is the gold standard treatment in ACL insufficient knees. A stable knee is paramount, especially in the young and active population who seek to return to their high activity level prior to incurring the injury. Preventing secondary degeneration and increased risk of meniscal tears in the knee after the ACL rupture are also goals of the treatment [1–3].

Evolution of the ACL reconstruction is still progressing, since the incidence of ACL ruptures is on the rise [4]. A wide range of graft fixation techniques is available when replacing the ruptured ACL. The modality of fixation the autograft using interference screws is the most popular fixation technique and has shown good results after long-term follow-up of up to 18 years [5]. The press-fit fixation offers the advantages of lower costs, absence of hardware-related complications, direct bone-to-bone healing, absence of metal artifacts on imaging and ease of revision surgery if necessary. Hertel was the first to develop and describe the femoral press-fit fixation technique in 1987. He then presented a tibial press-fit fixation in 1989 [6]. Boszotta and colleagues described the procedure using an arthroscopic approach in 1997 [7]. Several authors adopted this technique and reported good results on long-term follow-up [8–13].

In ACL reconstruction, utilizing an autograft tendon is the popular modality of choice to replace the original ACL. Appropriate graft selection remains a controversial topic and is largely based on the surgeon's experience and preference. The use of a bone–patellar tendon–bone (BPTB) autograft in reconstructing the ACL has been accepted as a gold standard for a long time. Results up to 18 years reported adequate stability, good knee function in sports, as well as a low recurrence rate [5, 14–16]. The use of a quadriceps tendon–patellar bone (QTB) autograft in ACL reconstruction is relatively less popular but is gaining momentum. Recent studies revealed good functional results and low failure rates [17], also those using press-fit fixation with a follow-up of up to 7.5 years [18]. Studies that report results 10 years after ACL reconstruction with the QTB autograft are lacking.

Both BPTB and QTP autografts offer the advantage of direct bone-to-bone integration of the graft versus the soft tissue to bone healing in the setting of ACL reconstruction with the hamstring autograft. Known disadvantages after harvest of the BPTB autograft include the risk of a patellar tendon tear or a patellar fracture and the increased donor site morbidity with associated anterior knee pain. Similar disadvantages are conceivable for the QTB graft. The authors are aware of few isolated cases of patients with a quadriceps tendon rupture or a patella fracture after the harvest of the QTB autograft. Some patients also report pain at the donor site [18, 19]. In a recent meta-analysis, Riaz et al. reviewed five studies comparing results of ACL reconstruction with BPTB and QTB autografts that included a total of 806 patients [20]. With a follow-up duration of 1 year, an equivalency with respect to knee stability and graft survival was demonstrated, yet an increased donor site morbidity was seen in patients who received a BPTB autograft.

The purpose of this study is to report and compare the 10-year results of ACL reconstruction with the BPTB versus QTB autografts using a press-fit fixation technique. Furthermore, a comparison with the short-term (1-year) results of the same cohort was done. Based on our previous unsystematic clinical observations, we hypothesized that the QTB autograft provides almost equal results in terms of stability and function of the knee joint, but the donor site morbidity would be lower than that of the BPTB graft.

## Materials and methods

This study was carried out as a prospective randomized study. The group assignment was carried out preoperatively by blockwise randomization using an urn model. The two surgical methods for ACL reconstruction using the two different autografts were very similar. Both transplants were anchored using a press-fit technique without the use of any hardware such as screws or buttons. The surgical techniques of this press-fit fixation using the QTB and BPTB autografts were accurately described in the published literature [7, 21]. All operations were performed in a single center by one senior surgeon (JH) who had an experience with over 1000 ACL reconstructions using BPTB autografts and over 150 ACL reconstructions using QTB autografts. The major components of the postoperative protocol were as follows: partial weight bearing for the first 5 days. Starting isometric muscle training and active exercises on the first day after the operation. Full knee joint extension should be achieved quickly, while flexion was limited to 90° during the first 14 days. No orthosis was used except in cases of concurrent meniscus or collateral ligament repair. Running was permitted 3 months after the operation. Contact sports was avoided 6–9 months after the operation.

The inclusion criteria included primary ACL rupture and an activity level with a minimum score of 6 on the Tegner scale [22]. Exclusion critieria included ACL re-rupture, concomitant instability of the PCL or lateral instability, the need for subtotal meniscal resection, presence of knee osteoarthritis stage 3 or 4 according to Kellgren–Lawrence score [23] and cartilage lesions greater than grade 2 in the Outerbridge classification.

The data were collected prospectively. A first examination was carried out preoperatively. The inclusion and exclusion criteria were checked pre- and intraoperatively. Further examinations were planned annually but did not take place between the 2nd and 9th year after the operation due to the lack of an investigator, so that the examinations were only carried out 1 year postoperatively and again after 10 years.

Subjective functional outcome was evaluated using the Lysholm score and the international Knee Documentation Committee score (IKDC 2000) [22, 24, 25]. The activity level on follow-up was scored using the Tegner scale [22]. Patient satisfaction was scored on a VAS scale from 0–10, with 10 being perfectly satisfied and 0 being dissatisfied with the result [26]. All intra- and postoperative complications as well as the need for reoperations were reported. The instrumental measurement of the anterior–posterior stability was carried out using the KT-1000™ Knee Ligament Arthrometer^®^ manufactured by MEDmetric^®^ Corporation (https://www.medicalproductguide.com/companies/1364/medmetric_corp, MEDmetric^®^ Corporation, 7542 Trade Street, San Diego, CA 92121; Patent No. 4,583,555) with a tension force of 134 N. The degree of knee degeneration was evaluated according to Kellgren–Lawrence classification [23]. Assessment of the tunnels involved X-rays of the knee taken in two planes (posterior–anterior/lateral view at 30° flexion standing).

This study has been approved by the appropriate ethics committee (Registration Number: S-122-98) and has, therefore, been performed in accordance with the ethical standards laid down in the 1964 Declaration of Helsinki. All patients gave their informed consent prior to their inclusion in the study.

### Statistical analysis

The entire data input and evaluation were carried out using the program IBM^®^ SPSS^®^ version 24. The normal distribution test was performed by the Kolmogorov–Smirnoff, respectively, by the Shapiro–Wilk test and the variance homogeneity by the Levine test. For the comparative statistics of the two groups, statistical significance was calculated for the metric scaled variables according to distribution and variance homogeneity by the *t* test, Mann–Whitney *U* test or Chi-quadrat test. For all the tests used, a *p* value of less than 0.05 was considered significant.

## Results

In total, 60 patients were included in the study and were preoperatively examined. During the procedures, no changes of the planned operation occurred after the preoperative randomization process took place. The press-fit fixation using the corresponding transplants was executed in all patients. Thirty patients received an ACL reconstruction with a BPTB autograft and 30 patients received an ACL reconstruction with a QTB autograft. The mean age at the time of surgery was 32 ± 7.5 years. The demographic data of the two groups showed no significant difference. Due to the exclusion criteria mentioned, accompanying injuries were only found in a few patients (20% of the total group). The frequency of subjects with accompanying injuries did not differ statistically between the two groups (*p* = 0.519, Chi-quadrat test). Details are represented in Table 1.

**Table 1:** Demographic data of the two groups: patients with quadriceps tendon–patellar bone autograft (= QTB group) and with bone–patellar tendon–bone autograft (= BPTB group) in comparison

*N* = 60	QTB group (*n* = 30)	BPTB group (*n* = 30)	Statistical significance test used
Sex: male/female	17/13 57%/43%	17/13 57%/43%	*p* = 1.000 Chi-quadrat test
Tegner score preinjury	Median 7 Range 6–9	Median 7 Range 6–9	*p* = 0.938 Mann–Whitney *U* test
Injured knee: right/left	18/12 60%/40%	12/18 40%/60%	*p* = 0.273 Chi-quadrat test
Interval injury surgery	Mean 1.8 $\pm$ 3.12 Range 27 days–11.92 years	Mean 1.4 $\pm$ 2.43 Range 26 days–10.08 years	*p* = 0.518 Unpaired *t* test
Age on operation day (years)	Mean 30.5 $\pm$ 7.8 Range 18–49	Mean 30.6 $\pm$ 7.5 Range 18–48	*p* = 0.960 Unpaired *t* test
Duration of surgery (minutes)	Mean 62 $\pm$ 9.3 Range 50–95	Mean 64 $\pm$ 15.3 Range 40–105	*p* = 0.544 Unpaired *t* test
Accompanying injuries (multiple choices possible)	7 (23%)	5 (17%)	*p* = 0.519 Chi-quadrat test
Meniscal injuries	4	3	
Cartilage lessons	2	3	
Inner ligament injuries	3	1	
1-year follow-up	*n* = 28	*n* = 28	
Interval operation: 1-year follow-up (days)	Mean 378 $\pm$ 15.72 Range 341–407	Mean 371 $\pm$ 22.09 Range 335–419	*p* = 0.176 Unpaired *t* test
10-year follow-up	*n* = 21	*n* = 22	
Interval operation: 10-year follow-up (years)	Mean 10.3 $\pm$ 0.25 Range 10.03–10.79	Mean 10.2 $\pm$ 0.24 Range 10.02–10.96	*p* = 0.284 Unpaired *t* test

One patient in the BPTB group suffered a re-rupture while dancing 7 months after the operation. His knee was reoperated on 3 months later with a hamstring autograft. Thereafter, he no longer wanted to participate in the planned follow-up examinations of the study. Over the course of the trial, two patients from each group were excluded due to an ACL rupture on the contralateral side. At 1 year postoperatively (mean 12.2 ± 1.9 months, range 10–14 months) 56 patients (93%) and after 10 years (mean 10.3 ± 0.25 years, range 10–11 years) 43 patients (72%) were followed-up clinically, functionally and radiologically.

**Fig. 1:**
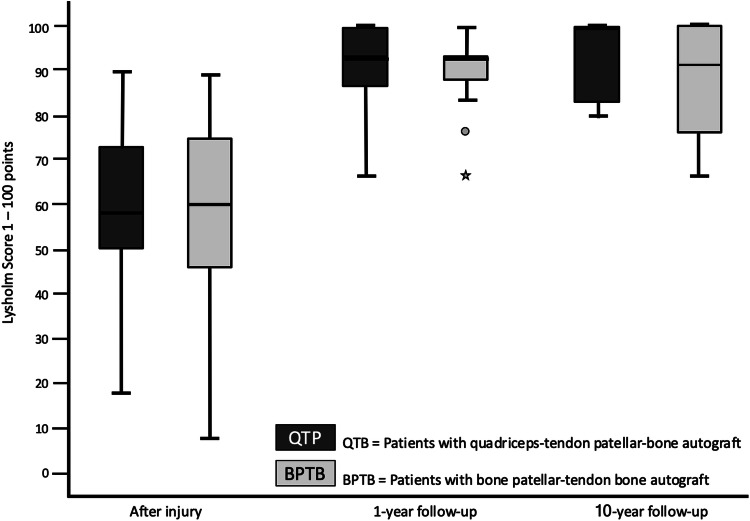
The Lysholm score in patients with quadriceps tendon–patellar bone autograft (= QTB group) and with bone–patellar tendon–bone autograft (= BPTB group) showed no significant difference between the groups at the following three points in time: after the injury and before surgery (*N* = 60, *p* = 0.612), 1 year postoperatively (*N* = 56, *p* = 0.834) and 10 years postoperatively (*N* = 43, *p* = 0.844)

**Fig. 2:**
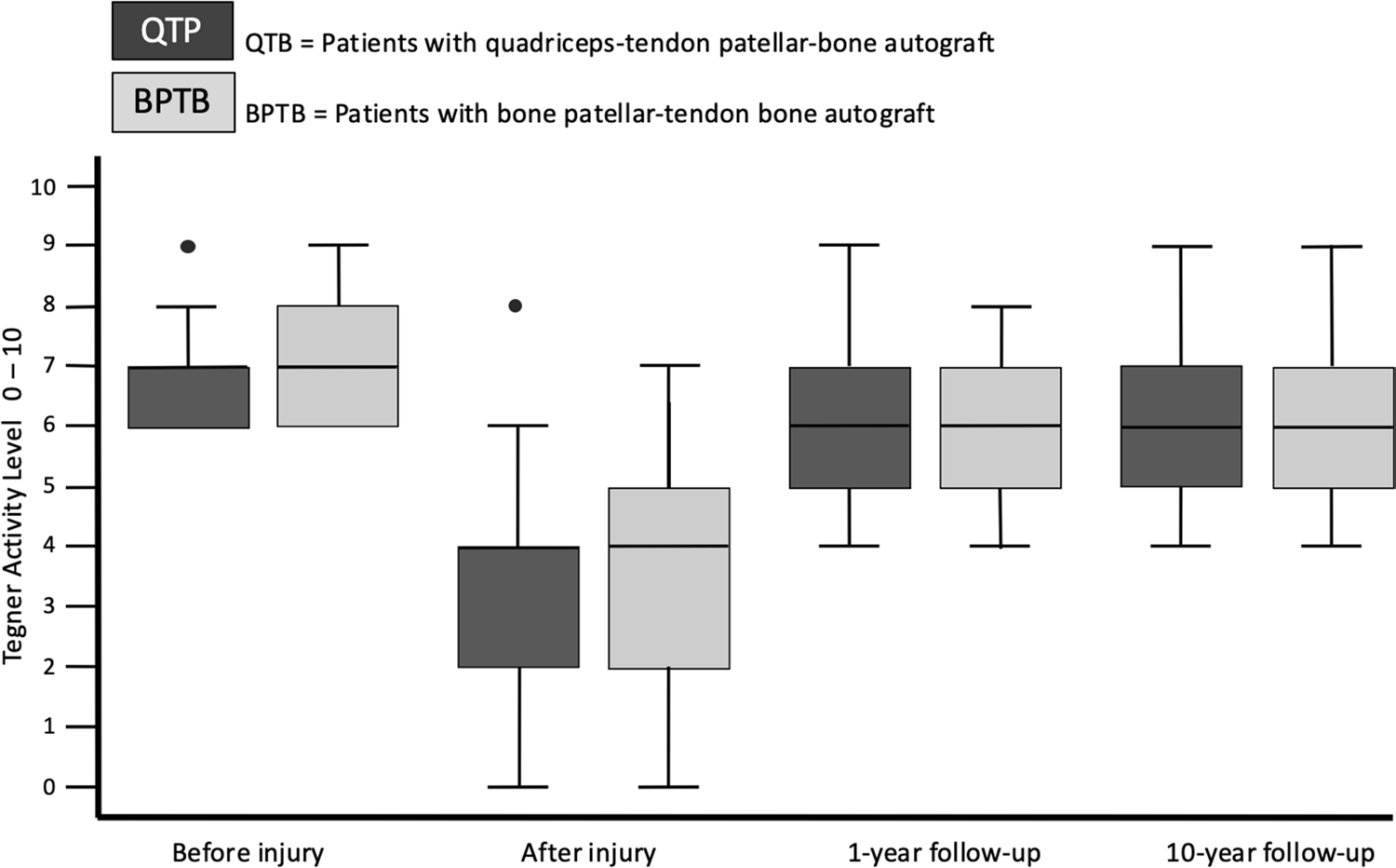
The Tegner score in patients with quadriceps tendon–patellar bone autograft (= QTB group) and with bone–patellar tendon–bone autograft (= BPTB group) showed no significant difference between the groups at the following four points in time: preinjury (*N* = 60, *p* = 0.938), after the injury and before surgery (*N* = 60, *p* = 0.273), 1 year postoperatively (*N* = 56, *p* = 0.518) and 10 years postoperatively (*N* = 43, *p* = 0.960)

**Fig. 3:**
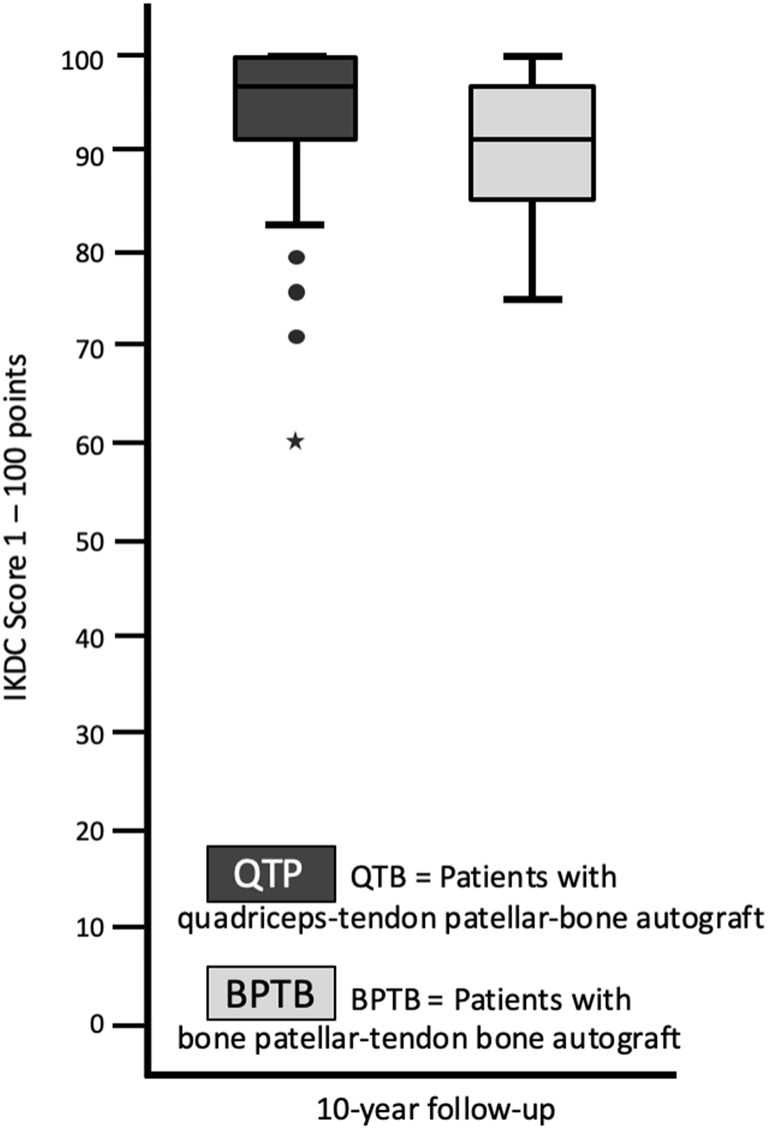
The IKDC score in patients with quadriceps tendon–patellar bone autograft (= QTB group) and with bone–patellar tendon–bone autograft (= BPTB group) showed no significant difference between the groups 10 years postoperatively (*N* = 43, *p* = 0.653)

**Fig. 4:**
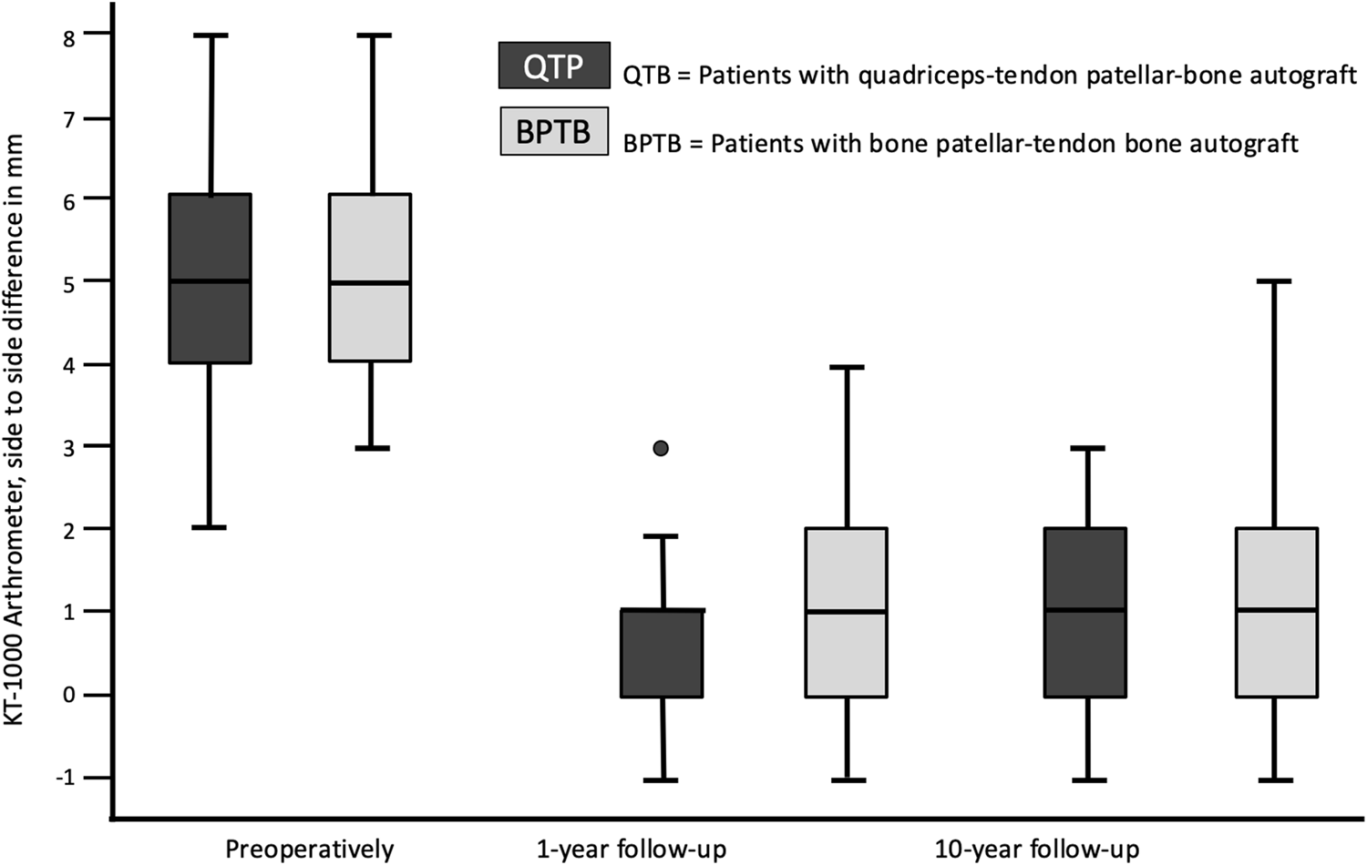
The KT-1000 arthrometer measurement in patients with quadriceps tendon–patellar bone autograft (= QTB group) and with bone–patellar tendon–bone autograft (= BPTB group) showed no significant difference between the groups at following three points in time: after the injury and before surgery (*N* = 60, *p* = 0.834), 1 year postoperatively (*N* = 56, *p* = 0.518) and 10 years postoperatively (*N* = 43, *p* = 0.235)

The preoperative Lysholm score for subjective evaluation of knee function showed good or very good results in only 5 (= 8%) patients. After 1 year, 88% and after 10 years, 90% of patients scored very good and good results (10 year result: mean 99 ± 7.1, range 74 to 100 points) (Fig. 1). The activity level using the Tegner scale had a high median score of 7 for all patients before incurring the injury. After having the injury but before the operation, the median score was 4 (range 0–8). On the 1- and 10-year follow-up, patients scored a median of 6 (range 4–9). 64% of all patients returned to at least the same level of activity they had before the injury but 36% couldn’t reach their preinjury level (Fig. 2). The IKDC score was reported on final follow-up. A normal or almost normal IKDC score was reported by 84% of the patients (median 97 ± 9.5, range 60 to 100 points) (Fig. 3). Measurement of the anterior translation of the knee with the KT-1000 Arthrometer showed a preoperative anterior instability of the injured knee in 97% of all cases (80% over 5 mm side to side difference, 17% 4–5 mm, 0% 3 mm, 3% 0–2 mm). After 1 year and also after 10 years, 95% of all knees were evaluated as stable (3 or less mm side to side difference) and 5% showed a instability between 4 and 5 mm. (10 year result: mean 1.0 ± 1.2, range − 1 to + 5 mm) (Fig. 4). Significant degeneration was radiologically detected in one patient per group. No tunnel widening was seen in any patient. Ninety-seven percent of all patients were very satisfied on final follow-up.

The statistical analysis did not show any significant differences between the two groups at any time (Table 2). The statistical comparison between the 10- and 1-year results did not show any significant difference: Tegner score (*p* = 0.645), Lysholm score (*p* = 0.298), KT-1000 arthrometer stability measurement (*p* = 0.235).

**Table 2:** Statistical results of the functional scores (IKDC = International Knee Documentation Committee Subjective Knee Form), the reported satisfaction with the procedure (VAS = Visual Analogue Scale) and the knee stability (KT-1000 = side to side difference of the posterior–anterior translation in mm using the KT-1000 Arthrometer). Patients with quadriceps tendon–patellar bone autograft (= QTB group) and with bone–patellar tendon–bone autograft (= BPTB group) in comparison

	QTB group	BPTB group	Statistical significance test used
Lysholm score			
Preoperative *N* = 60	Mean 59.7 ± 18.6 Range 18 to 90	Mean 56.9 ± 22.3 Range 8 to 89	*p* = 0.612 Unpaired *t* test
1-year follow-up *N* = 56	Mean 95.9 ± 6.1 Range 77 to 100	Mean 96.2 ± 5.3 Range 73 to 100	*p* = 0.834 Unpaired *t* test
10-year follow-up *N* = 43	Mean 95.6 ± 7.8 Range 74 to 100	Mean 95.2 ± 6.6 Range 75 to 100	*p* = 0.844 Unpaired *t* test
Tegner score			
Preinjury *N* = 60	Median 7 Range 6 to 9	Median 7 Range 6 to 9	*p* = 0.938 Mann–Whitney *U* test
Preoperative *N* = 60	Median 4 Range 0 to 8	Median 4 Range 0 to 7	*p* = 0.273 Mann–Whitney *U* test
1-year follow-up N = 56	Median 6 Range 4 to 9	Median 6 Range 4 to 8	*p* = 0.518 Mann–Whitney *U* test
10-year follow-up *N* = 43	Median 6 Range 4 to 9	Median 6 Range 4 to 9	*p* = 0.960 Mann–Whitney *U* test
IKDC score			
10-year follow-up *N* = 43	Mean 92 ± 11.5 Range 60 to 100	Mean 91 ± 7.3 Range 75 to 100	*p* = 0.653 Unpaired *t* test
Satisfaction (VAS 0–10)			
10-year follow-up *N* = 43	Median 10 Range 7 to 10	Median 10 Range 8 to 10	*p* = 0.284 Mann–Whitney *U* test
KT-1000			
Preoperative *N* = 60	Median 5 Mean 5.00 ± 1.4 Range + 2 to + 8	Median 5 Mean 5.33 ± 1.4 Range + 3 to + 8	*p* = 0.362 Unpaired *t* test
1-year follow-up *N* = 56	Median 1 Mean 0.70 ± 0.9 Range − 1 to + 3	Median 1 Mean 1.13 ± 1.3 Range − 1 to + 4	*p* = 0.142 Unpaired *t* test
10-year follow-up *N* = 43	Median 1 Mean 1.00 ± 1.095 Range − 1 to + 3	Median 1 Mean 1.05 ± 1.36 Range − 1 to + 5	*p* = 0.235 Unpaired *t* test

**Fig. 5:**
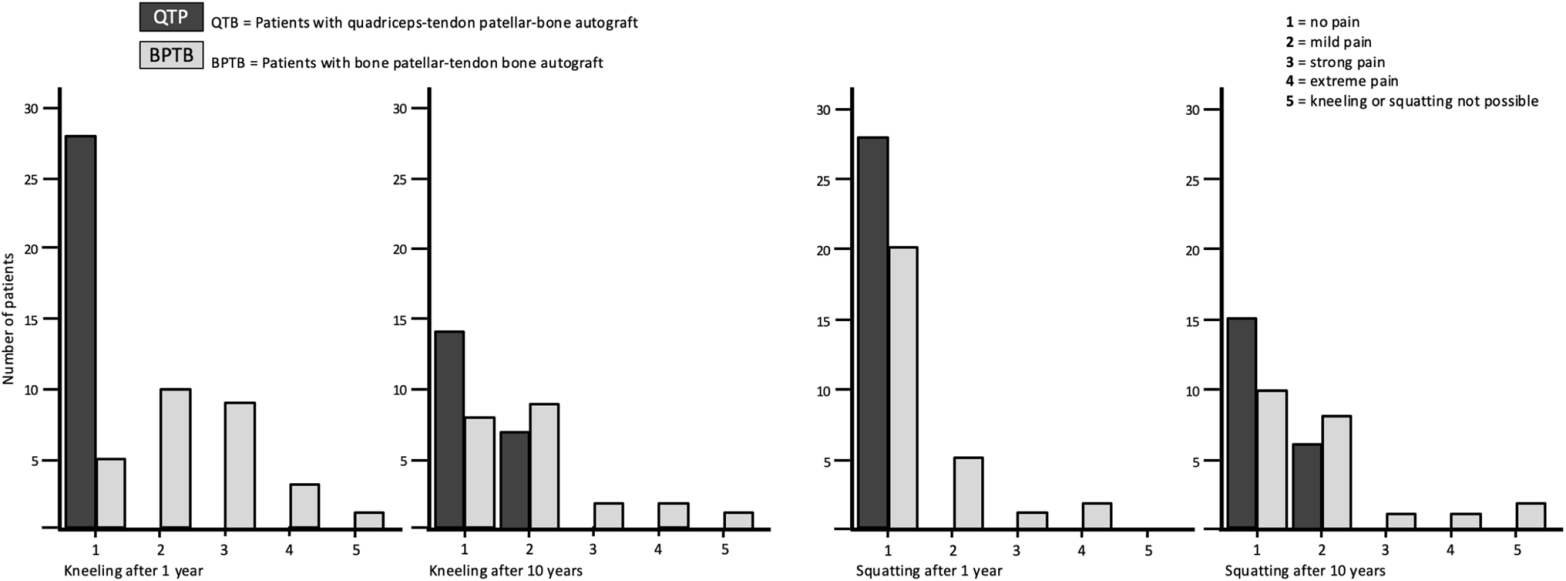
Donor site morbidity: The prevalence of postoperative pain during kneeling and squatting was significant higher in patients with bone–patellar tendon–bone autograft (= BPTB group) than in patients with quadriceps tendon–patellar bone autograft (= QTB group) at the following two points in time: 1 year postoperatively (*N* = 56, kneeling pain *p* < 0.001, squatting pain *p* = 0.003) and 10 years postoperatively (*N* = 43, kneeling pain *p* = 0.019, squatting pain *p* = 0.046)

The only significant difference between the groups was evident in terms of increased donor site morbidity in the BPTB group (IKDC score, subjective assessment form of the knee, questions 9c and 9d) (Fig. 5). The prevalence of postoperative pain during kneeling was higher at the BPTB than the QTP group after 1 year (BPTB 92%, QTP 0%, *p* < 0.001) and after 10 years (BPTB 64%: 41% mild, 23% severe, QTP 33%: all mild, *p* = 0.019). The prevalence of postoperative pain during squatting was also higher at the BPTB than the QTP group after 1 year (BPTB 29%, QTP 0%, *p* = 0.003) and after 10 years (BPTB 55%: 36% mild, 18% severe, QTB 29%: all mild, *p* = 0.046).

The influence of accompanying injuries (at the time of the operation) on the results was analyzed. The comparison of patients with (+ AI) and without accompanying injuries (− AI) showed a tendency for worse results after 10 years for patients with concomitant injuries without this difference becoming statistically significant for all parameters tested (Mann–Whitney *U* test). Lysholm score, mean: + AI 91 ± 10.0 points, − AI 95 ± 8,6 points, *p* = 0.123; IKDC score, mean: + AI 89 ± 11.9 points, − AI 92 ± 9.0 points, *p* = 0.643; Tegner scale, median (range): + AI 6 (4–7), − AI 6 (4–9), *p* = 0.646; KT 1000 Arthrometer, mean (range): + AI 1.2 ± 1.0 mm (0–3 mm), − AI 1.1 ± 1.4 mm (− 1 to 5 mm), *p* = 0.570; pain during kneeling: + AI 56% with pain, − AI 50% with pain, *p* = 0.632; pain during squatting: + AI 56% with pain, − AI 39% with pain, *p* = 0.387.

During the first year, three patients from the BPTB group and one patient from the QTB group were operated on arthroscopically due to developing cyclops syndrome which led to limited knee extension. All reoperated patients reached full range of motion of the knee postoperatively. After the first year, one patient from the BPTB group and two patients from the QTB group received arthroscopic meniscal repair. Other complications such as graft dislocation, patellar fracture, infection or tendon rupture did not occur in either group.

## Discussion

The most important finding of this study is that the QTB group achieved a comparable stability and return-to-sport level in athletes on 10-year follow-up as the BPTB group, while having the advantage of a significantly less donor site morbidity.

A recent meta-analysis reported BPTB autografts to provide superior rotational knee stability in comparison with hamstring autografts, albeit with higher donor site complication rate in the form of anterior knee pain, especially during kneeling [27, 28]. This pain during kneeling after BPTB ACL reconstruction is recurrently reported in the medical literature and can lead to patient dissatisfaction [29, 30]. The increased anterior knee pain after harvest of the BPTB autograft seems to be a result of the autograft extraction itself, rather than the reconstructive element of the surgery, as using contralateral BPTB autografts results in anterior knee pain and kneeling difficulties being shifted from the injured to the healthy side [31]. Correlations between these adverse effects and disturbance of anterior knee sensitivity were demonstrated [32, 33]. Damage of the infrapatellar nerve seems to play a main role. But since its course and branching are unspecific, efforts for its conservation are perhaps not clinically beneficial. In the presented study, patient satisfaction was not affected, but the complaint was reported. The results of this study show that when using a BPTB allograft, pain and problems with kneeling and squatting must be expected over the long term. However, when using the QTB allograft, these problems occur significantly less frequently and are usually classified as mild as opposed to the BPTB autograft. There are many sports where taking these results into account is beneficial [34]. A good example of this is Judo. Some surgeons at Judoka reject the use of a hamstring autograft since this weakens the active medial stabilization of the knee. Kneeling is especially common in Judo and pain caused by a transplant removal can be very troublesome.

The analysis of accompanying injuries in this study showed only a tendency towards poorer results after 10 years. The groups were relatively similar because severe accompanying injuries were excluded. Slight accompanying injuries were equally common in the two groups. Therefore, it cannot be assumed that the negative effect had a significant impact on the comparison of the groups.

The initial use of the QTB autograft in primary ACL reconstruction was questionable since a number of complications was reported. These mainly included a postoperatively detectable pivot shift tests and weakness in knee extension [35]. The reason for the occurance of these complications is not clear. The fact that the surgeons had a limited experience with this procedure could be a plausible explanation. Recent studies have, however, debunked these findings, showing the QTB autograft as a decent contender in primary ACL reconstructive surgery [18, 21, 36, 37]. A follow-up of 7.5 years showed excellent subjective and objective results as well as a relatively low donor site morbidity rate [18]. Surgeons were rather reserved when it came to harvesting a QTB autograft in the past, because rupture or weakening of the muscle was feared. Instead, the published literature suggests comparable quadriceps recovery when either QTB or BPTB autografts are used [38]. This is especially important in athletes since fast muscle recovery is crucial for resuming a high level of activity.

The clinical results regarding the knee function do not appear to differ significantly between the grafts on long-term follow-up. This is seemingly a result of the intraarticular ligamentization process that all grafts undergo after reconstruction. This remodeling eventually leads to a ligamentous “ACL-like” structure which histologically resembles a normal ACL. Only ultrastructural differences regarding collagen fibril distribution persist [39]. The ligamentization process has been mainly described in the patellar tendon graft and the hamstring tendon graft. Studies that describe the ligamentization process of the QTB autograft are lacking [40, 41].

Graft fixation with intereference screws is one of the most popular and established methods in ACL reconstructive surgery. Strength and stiffness of the autograft are crucial for resisting slippage under cyclic loading in the initial postoperative phase. Stability of the graft without the use of hardware was theoritically debatable. In biomechanical studies, however, the femoral press-fit fixation possessed adequate primary stability with ultimate load to failure at least equal to results for interference screws [13, 42]. The tibial hybrid fixation with a bone bridge and spongiosa filling also demonstrated the same tear strengths under experimental conditions as interference screw fixation [43]. This clinical study identified no complication in any patient which could be attributable to the fixation technique. No tunnel expansion occurred compared to reported cases using other fixation materials [44]. The press-fit fixation approach thus provides good stability while offering many advantages such as lower costs in the absence of hardware equipment, no hardware-related complications and direct bone-to-bone healing. Furthermore, revision surgery is made easier since drill tunnels were filled with bony material, one-sided revision in this case is possible without the need for spongioplasty.

The strengths of this study include its prospective nature, the equal randomizations of the two groups and the comprehensive follow-up examination. Additionally, all patients underwent the same procedures by one senior surgeon. The main weaknesses of this study is the loss to follow-up rate of more than 20% after 10 years.

## Conclusions

This study shows very good functional, clinical and radiological long-term results for both hardware-free methods of ACL reconstruction in patients with a high activity level. Results clinically confirm the safety of press-fit anchoring with consistent results even on the long term. The increased donor site morbidity when using the BPTB autograft compared to the QTB autograft is already known for methods with hardware fixation and has been confirmed in this study for the implant-free press-fit technique [20]. The use of QTB over BPTB autografts can be especially considered in patients who frequently exercise kneeling and squatting in sport or occupation.
